# The reliability of chest radiographs in predicting left atrial enlargement

**DOI:** 10.5830/CVJA-2010-028

**Published:** 2010

**Authors:** SJ Quinton, A Deffur, JA Ker, P Rheeder

**Affiliations:** Department of Internal Medicine, School of Medicine, University of Pretoria, and Steve Biko Academic Hospital, Pretoria, South Africa; Department of Internal Medicine, School of Medicine, University of Pretoria, and Steve Biko Academic Hospital, Pretoria, South Africa; Faculty of Health Sciences, University of Pretoria, Pretoria, South Africa; Department of Clinical Epidemiology, School of Medicine, University of Pretoria, Pretoria, South Africa

**Keywords:** sub-carinal angle, sub-angle distance, chest radiograph, left atrial size

## Abstract

**Introduction:**

Estimates of left atrial size in patients with suspected cardiac disease play an important role in predicting prognosis and events, as well as treatment decisions. Two methods are commonly used to estimate left atrial size: chest radiography and cardiac ultrasound. This study aims to determine the test characteristics by comparing the use of radiographs to cardiac ultrasound (the gold-standard test).

**Methods:**

Data from patients older than 18 years admitted to Steve Biko Academic Hospital during 2000–2003 who had both chest radiographs and cardiac ultrasound were included in this cross-sectional, retrospective analysis. Chest radiographs were classified into three quality classes, and the sub-carinal angle (SCA) and sub-angle distance (SAD) were measured twice in all available radiographs by two observers. Intra- and inter-observer variability (three methods) as well as the predictive value of the carinal angle and sub-angle distance measurements were determined using logistic regression (with left atrial enlargement – determined by ultrasound as comparator).

**Results:**

Data for 159 patients were available (154 cardiac ultrasounds and 178 chest radiographs). Intra-observer variability for chest radiograph measurements was low with almost perfect concordance (*p* = 0.000). Inter-observer variability was higher for supine radiographs. Using logistic regression, a linear model was identified which was statistically significant only for erect radiographs. While goodness-of-fit analysis showed that the model fits the data, performance characteristics were poor, with high sensitivity and low specificity, and an area under the ROC curve of 0.62–0.63, depending on type of radiograph and measurement (SCA or SAD). Linearity in the logit of the dependent variable was assessed, and found to be present at the extremes of SCA measurements for the supine radiograph data and in the first three quartiles for erect radiograph data. A non-linear model determined by fractional polynomial analysis did not perform significantly better than the original linear model. Cut-off values for the SCA of 72° and 84° (erect and supine radiographs, respectively) were found to give the best compromise between sensitivity and specificity. The corresponding cut-off values for SAD were 24.1 and 26.9 mm.

**Conclusion:**

Assessment of either SCA or SAD to determine left atrial size was equivalent and repeatable, both with the same observer and between two observers (less so for supine radiographs). While this measure was precise, it was found not to be very accurate. Therefore, chest radiographs are not reliable in predicting left atrial enlargement.

## Summary

Left atrial size can be useful in clinical medicine both as a prognostic marker and in the prediction of clinical events. Postoperative symptomatic improvement following surgery for aortic stenosis may be predicted using left atrial size.^1^ An echocardiographic study^2^ found a significant correlation between left atrial size and atrial natriuretic peptide levels after acute myocardial infarction both at 10 to 12 days post infarction as well as at six months. Based on a correlation coefficient of 0.70, it was concluded that the percentage change in the size of the left atrium could reliably predict the percentage change in atrial natriuretic peptide after an acute myocardial infarction.

In a long-term study of patients with dilated cardiomyopathy, echo-derived atrial dimension was found to be the major predictor of cardiac death and clinical outcome compared to other echocardiographic, clinical and haemodynamic parameters at time of entry into the study.^3^

Left atrial appendage area is increased in patients with atrial fibrillation,^4^ a known increased risk of cardioembolic stroke. An increased left atrial size also correlates with an increased risk of stroke in patients with sinus rhythm.^5^ In an ethnically mixed population, left atrial size is proportionate to the risk of ischaemic stroke.^6^

Left atrial size is an important determinant in the response to treatment of atrial fibrillation, such as cardioversion for atrial fibrillation where left atrial size decreased after restoration of sinus rhythm in all patients.^7^ Left atrial size may indicate which patients could benefit from anti-arrythmic therapy. Sinus rhythm can be maintained after cardioversion with left atrial sizes varying between 45 and 60 mm, and with greater left atrial dimensions, atrial fibrillation was likely to return.^8^

Biplane two-dimensional echo is accurate for determining left atrial size^9^ but is not widely available or affordable. Chest radiographs are often used to estimate left atrial size, being cheaper and more readily available, and clinical decisions are often based on this without regard to the accuracy and precision of this measurement.

Two previous publications^10^,^11^ investigated estimation of left atrial size by measuring the carinal angle and comparing that to echo findings. In the first study, 35 patients with enlarged left atria (> 4.5 cm), and 35 paired, age-matched patients with normal atria (< 4.0 cm) were selected as determined by echo. Interbronchial angle on chest radiographs (standard and supine portable films) was measured by a blinded observer using a goniometer. Left atrial size could be accurately predicted to be larger than 5.0 cm in diameter if the carinal angle was greater than 100° (*r* = 0.746 with *p* < 0.001), but the sample size of 63 patients may have been too small.

In the second study, the postero-anterior chest radiographs and echocardiographs of 108 clinically stable patients (53 men and 55 women) were respectively reviewed. Left atrial size correlated poorly with both interbronchial angle (*r* = 0.33) and sub-carinal angle (*r* = 0.25). An interbronchial angle of 76.4° and a sub-carinal angle of 65.4° were the best discriminators between patients with normal and those with enlarged left atrial dimensions (sensitivities: 63 and 51%, specificities: 63 and 66%, for interbronchial angle and sub-carinal angle, respectively) but no intra- or inter-observer variation were tested.

The aim of this study was to investigate the precision and accuracy of chest radiographs to predict left atrial enlargement in comparison to measurement by echocardiography.

## Methods

The study was conducted at Steve Biko Academic Hospital, a regional tertiary referral centre. The study was a cross-sectional retrospective analysis.

Patients older than 18 years admitted to the hospital between January 2000 and December 2003 who had had both echocardiography and chest radiography performed during the same admission were included in the analysis. The sampling frame was determined by accessing records at the ultrasound department and finding all those patients who had had echocardiography during the specified period. All radiographs of the identified patients were retrieved and assessed according to quality criteria, and only those patients with adequate radiographs were included in the final analysis.

Demographic data were collected on all patients. Left atrial size was determined with two-dimensional targeted M-mode echocardiography using a Vivid 3 General Electric ultrasound machine. Left atrial size was determined according to the American Society of Echocardiography (ASE) criteria.^12^

In determining left atrial size, the maximal dimension was measured from the parasternal long-axis view between the leading edge of the posterior aortic wall and the leading edge of the posterior wall of the left atria at end-systole. Left atrial size may be underestimated in the parasternal long-axis view because this chamber may enlarge longitudinally.^13^ Therefore, left atrial size was measured from two optical orthogonal views (four-chamber and two-chamber) as well, from the tip of the mitral valve to the posterior wall of the left atrium at end-systole, and the larger of the two values was used in the analysis.

Two independent observers assessed chest radiographs twice. Duplicate radiographs were removed from the analysis so that each cardiac ultrasound was matched with one radiograph (either supine or erect). Radiographs were read on a radiographic viewing box. The angle of divergence (α) of the first few centimetres of the inferior main-stem bronchial borders was measured using a protractor. The sub-angle distance (SAD) *x* (in mm) on the side opposite to the sub-carinal angle (SCA) α (alpha, in degrees) was measured 20 mm from the sub-carinal angle along the medial borders of the bronchi (*y*) using a tape measure.

The sample size calculation was performed using the program nQuery Advisor®. With α and power set at 5% and 90%, respectively, δ (expected proportion of subjects with enlarged left atrial size) estimated at 70%, K_0_ (hypothetical perfect agreement between two methods) chosen as 90%, and K_1_ (expected agreement between two methods) as 75%, the sample size was estimated at 106. Allowing for 10% of patients with echocardiograms not having traceable chest radiographs, and expecting 20% of carinal angles not to be clearly visible on chest radiographs, an initial sample of 150 was determined.

## Statistical analysis

Intra- and inter-observer agreement for the SCA and SAD were performed using Lin’s concordance correlation coefficient^14^ limits of agreement (Bland and Altman methodology^15^ and Bland and Altman plots). Logistic regression was used to determine the predictive value of the SCA and SAD for both erect and supine radiographs. First, a linear model was determined. This was followed by assessment of the fit of the model and its performance characteristics. Finally, to assess whether the dependent variable was linear in the logit, three methods, as proposed by Hosmer and Lemeshow,^16^ were used: lowess (locally weighted least squares) smoothing curves, design variables and fractional polynomials.

Cut-off values for the SCA or SAD that resulted in the best compromise between sensitivity and specificity were determined using receiver operating characteristic (ROC) curves.^17^ *P*-values < 0.05 were regarded as statistically significant for all comparisons. All calculations were performed using Intercooled Stata version 8.2.

## Results

Echocardiography and radiography data were available on 159 patients, 74 males and 85 females. The mean age of the study sample was 59 years (range 18–88 years). Five echocardiograms were incomplete as left atrial size was not measured, and therefore only 154 echocardiograms were included in the logistic regression analysis. As several patients had more than one chest radiograph taken, 178 chest radiographs were available for determination of intra- and inter-observer variability. Table 1 describes the radiographic quality of the X-rays used to determine left atrial size.

**Table 1.** Radiograph Characteristics

*Chest radiographs*	*Good*	*Fair*	*Poor*	*Total*
Supine	43	1	3	47
Erect	115	10	6	131
Total	158	11	9	178

Agreement of both SCA and SAD using observer 1 (intraobserver agreement) was near perfect, with Lin’s correlation coefficient ranging from 0.98 to 0.99 (*p* = 0.000) across all categories of radiographs (erect and supine, good, fair and poor quality). Agreement of both SCA and SAD using observer 2 (intra-observer agreement) was fair, with Lin’s correlation coefficient ranging from 0.92 to 0.99 (*p* = 0.000) across all categories of radiographs (erect and supine, good, fair and poor quality).

The agreement between observer 1 and observer 2 (interobserver agreement) regarding the measured mean SCA and mean SAD was poor to fair and varied between 0.74 and 0.93 for different combinations of radiograph types. All results were statistically significant.

Eighty-four of 154 echocardiograms showed left atrial dimensions exceeding 40 mm, and were classified as ‘enlarged’. The prevalence of enlarged left atrium as determined by echocardiography (the gold standard) was therefore 55%.

Logistic regression with either the SCA or the SAD yielded the model and its performance characteristics are shown in Table 2. Only good-quality radiographs and the observations from observer 1 were used.

**Table 2.** Logistic Regression Model With Performance Characteristics

*Good-quality radiographs*	*Erect*		*Supine*	
Mean of observer 1	SCA	SAD	SCA	SAD
Number of observations	102	102	37	37
Coefficient (SE)	0.02 (0.01)	0.09 (0.04)	0.03 (0.02)	0.10 (0.07)
LR χ^2^	4.41	5.12	3.16	3.41
OR	1.02	1.09	1.03	1.1
*p*-value	0.04	0.02	0.08	0.06
95% CI	–1.05	1.01–1.19	0.99–1.07	0.95–1.27
GOF	0.43	0.15	0.38	0.21
Sensitivity (%)	82.76	82.76	80.00	80.00
Specificity (%)	34.09	36.36	52.94	47.06
PPV (%)	62.34	63.16	66.67	64.00
NPV (%)	60.00	61.54	69.23	66.67
AUC	0.62	0.63	0.62	0.63

SCA: sub-carinal angle, SAD: sub-angle distance, SE: standard error, LR: likelihood ratio, OR: odds ratio, CI: confidence interval, GOF: goodness-of-fit, PPV: positive predictive value, NPV: negative predictive value, AUC: area under the curve.

Linearity of the dependent variable (SCA or SAD) for both erect and supine radiographs in the logit of the independent variable was assessed using three methods. This was done to confirm that binomial logistic regression was the appropriate method of analysis.

**Fig. 1.**
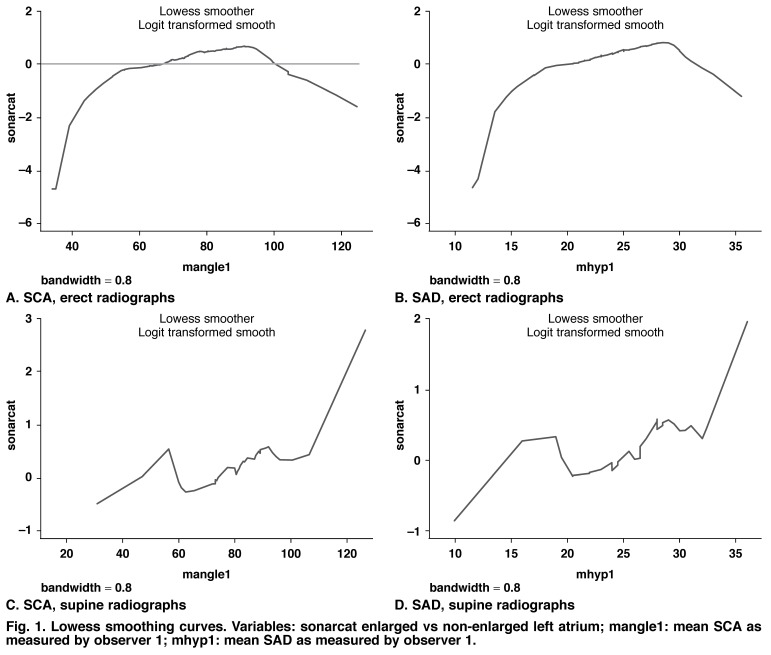
Lowess smoothing curves. Variables: sonarcat enlarged vs non-enlarged left atrium; mangle1: mean SCA as measured by observer 1; mhyp1: mean SAD as measured by observer 1.

**Fig. 2.**
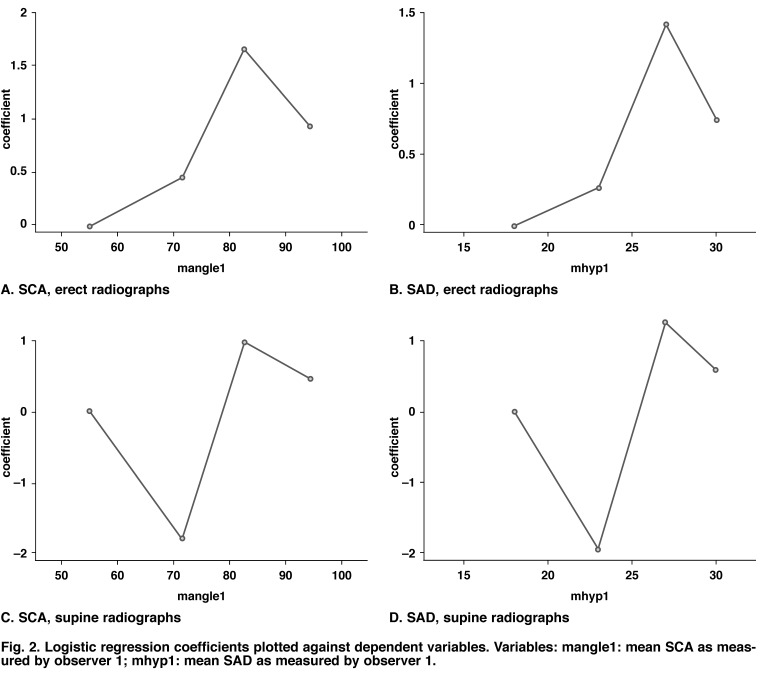
Logistic regression coefficients plotted against dependent variables. Variables: mangle1: mean SCA as measured by observer 1; mhyp1: mean SAD as measured by observer 1.

• Lowess smoothing curves showed that linearity varied over the interval of the dependent variable. It was present throughout in the case of erect radiographs, but in the case of supine radiographs only at the extremes of measurement, corresponding to small and large angles/SADs, respectively. This is illustrated in Fig. 1.

• Logistic regression coefficients were plotted against the approximate quartile midpoints of dependent variables as shown in Fig. 2. The results of the quartile analysis as plotted show a definite deviation from linearity in the fourth quartile throughout all permutations of radiograph types and variables used in analysis. By contrast, the results suggest linearity in the logit for both SCA and SAD in the first three quartiles for erect radiographs.

• Fractional polynomial model comparisons showed that the best non-linear transformations were not significantly different from the linear model. Therefore, the fractional polynomial analysis supported treating both variables as linear in the logit in general, with one exception: a significant *p*-value of 0.04 for the variable SCA suggested that the fit of the model might be improved if the variable was transformed by its inverse square. This in turn suggested that the use of the transformed variable in the logistic regression analysis might result in a superior model.

**Fig. 3.**
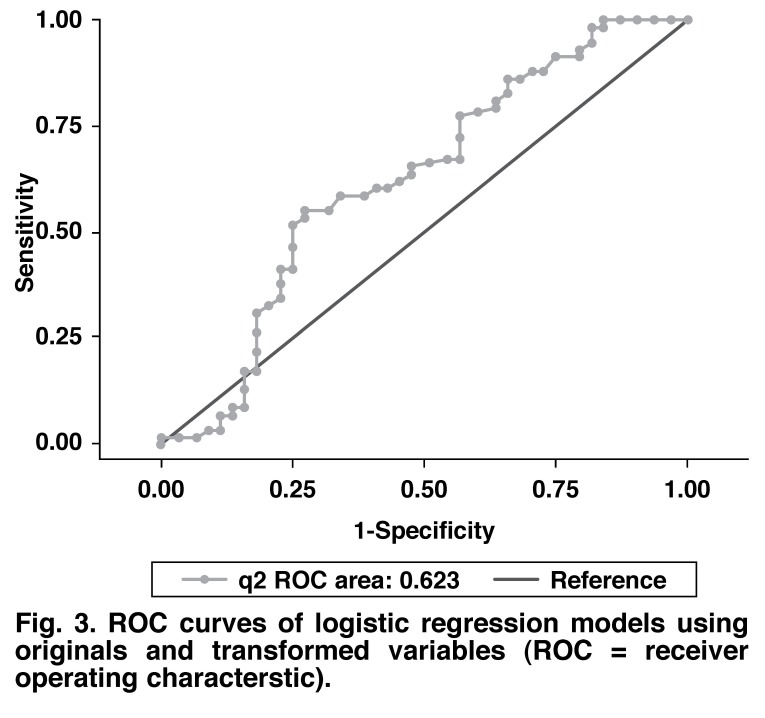
ROC curves of logistic regression models using originals and transformed variables (RO C = receiver operating characterstic).

The logistic regression results of the transformed variable, when compared to the original variable, seemed similar. This similarity was borne out in the perfect overlap of the ROC curves of the two models (Fig. 3). The discriminating value of the diagnostic test did not improve by using a transformed variable.

A diagnostic test is useful if it has both high sensitivity and specificity. Tests that measure continuous or categorical data can distinguish between normal and abnormal, based on cutoff values. These values can in principle be arbitrarily chosen. In practice, however, those values that offer the best trade off between sensitivity and specificity are usually employed. Using the linear model above, cut-off values differentiating between a non-enlarged and an enlarged left atrium based on chest radiographs were determined using ROC curves. The results are shown in Table 3.

**Table 3.** Cut-Off Values To Determine Left Atrial Enlargement

		*Erect radiographs*	*Supine radiographs*
SCA	Cut-off value	> 71.72°	> 83.525°
	Sensitivity (%)	61.02 (47.44–73.45)	47.62 (25.71–70.22)
	Specificity (%)	56.86 (42.25–70.65)	63.16 (38.36–83.71)
SAD	Cut-off value	> 24.1 mm	> 26.9 mm
	Sensitivity (%)	59.32 (45.75–71.93)	57.14 (34.02–78.18)
	Specificity (%)	60.78 (46.11–74.16)	63.16 (38.36–83.71)

SCA: sub-carinal angle, SAD: sub-angle distance.

## Discussion

This study explored the intra- and inter-observer variability of chest radiograph measurements of left atrial size and aimed to determine the diagnostic utility of chest radiographs compared to echocardiography in predicting left atrial size. Data from 159 patients were sampled. Five patients had to be excluded from the final analysis due to undocumented left atrial size on echocardiography.

Intra-observer agreement of measures of left atrial size was excellent for both observers. Lin’s correlation coefficient ranged from 0.98 to 0.99 for observer 1 and 0.92 to 0.99 for observer 2 across all categories of radiographs. This implied that the methods (using the SCA or SAD) were very precise. Inter-observer agreement between observer 1 and observer 2 ranged between 0.88 and 0.93 across all categories of radiographs except for supine radiographs in isolation, which yielded coefficients between 0.74 and 0.88. This underscores the precision (repeatability) of measuring SCAs or SADs.

The linear model obtained using logistic regression demonstrated that only erect chest radiographs were useful in predicting whether the left atrium was enlarged (all *p* < 0.05), as supine chest radiographs did not yield statistically significant results, with *p*-values of 0.08 and 0.06 for SCA and SAD, respectively. This could be due to the small number of supine radiographs.

Goodness-of-fit results for the above model, varying between 0.15 and 0.43 (all *p* > 0.05), showed that the model was a reasonable fit for both erect and supine radiographs using either variable (SCA or SAD), which implies that the model effectively described the outcome variable.

Although the sensitivity of all four categories (erect vs supine radiographs using SCA vs SAD) was consistently above 80%, the specificity was poor (< 53%), so that a normal value would rule out enlargement of the left atrium. Positive and negative predictive values (as well as the area under the ROC curve) were close to 50% (± 60%) implying that either a positive or negative result was hardly better than a random guess in predicting left atrial enlargement. This in effect means that a rule-out strategy (as suggested by the high sensitivity) using these variables (SCA/SAD) on their own was not feasible as the model’s ability to discriminate between enlarged and normal left atria was poor.

The visual representation of the relationship between the actual left atrial size category and the estimate (SCA and SAD) as portrayed in the Lowess smoothing curves (Fig. 1) suggests a linear relationship only at the extremes of SCA (or equivalent SAD). Between angles of 50° (18 mm) and 110° (32 mm), the prediction of left atrial enlargement was unreliable, especially for supine radiographs.

The results of the quartile analysis as plotted in Fig. 2 show a definite deviation from linearity in the fourth quartile throughout all permutations of radiograph types and variables used in the analysis. By contrast, the results suggest linearity in the logit for both SCA and SAD in the first three quartiles for erect radiographs.

The last measure of linearity in the logit (fractional polynomials) suggested a non-linear model using a transformed SCA (its inverse square). As this model’s performance did not differ from the linear one (Fig. 3), it may be concluded that no non-linear transformation can in fact improve the predictive value of the model.

The model may therefore be regarded as linear in the logit of the parameters, using either dependent variable (i.e. SCA or SAD). This means that logistic regression is the appropriate method for deriving a prediction rule for left atrial size category (enlarged/not enlarged) using radiograph-derived left atrial size estimates.

Optimal cut-off values (Table 3) for the SCA and SAD of both erect and supine radiographs revealed surprising results. The commonly used cut-off value for the SCA in clinical practice thought to indicate an enlarged left atrium is 90°. By contrast, this study found the cut-off points to be 72° for erect and 84° for supine films. In addition to this, the SAD measure seemed to be interchangeable with the SCA measurement throughout the analysis. Corresponding cut-off values for SAD for erect and supine films were 24.1 and 26.9 mm, respectively.

There is a considerable degree of overlap in the range of bifurcation angles measured in patients with normal and enlarged left atria, as confirmed by a CT study of tracheal bifurcation angles.^18^

## Conclusion

Both SCA and SAD can be used interchangeably on erect chest radiographs of good quality to predict left atrial enlargement with great precision but poor accuracy. Cut-off values below the traditionally used 90° were found to predict left atrial enlargement with improved diagnostic characteristics. The corresponding SAD appeared to have slightly better diagnostic discriminatory capability regarding test sensitivity, specificity and corresponding confidence intervals surrounding those measures

The use of chest radiographs in predicting left atrial enlargement is not recommended due to low sensitivity and specificity of the determined cut-off values. Chest radiographs have poor diagnostic utility in predicting left atrial enlargement. Echocardiography remains the preferred method for determining left atrial size.
